# Knowledge, Attitudes, and Practices of Moroccan Community Rheumatologists’ Regarding the Management of Non-radiographic Axial Spondyloarthritis: A National Cross-Sectional Study

**DOI:** 10.7759/cureus.61162

**Published:** 2024-05-27

**Authors:** Fatine Kronbi, Hanan Rkain, Nada Benzine, Samya Ez-zaoui, Radouane Abouqal, Jihane Belayachi, Najia Hajjaj-Hassouni, Latifa Tahiri, Fadoua Allali

**Affiliations:** 1 Rheumatology, Ayachi Hospital, Ibn Sina Hospital Center, Faculty of Medicine and Pharmacy, Mohammed V University, Rabat, MAR; 2 Exercise Physiology and Autonomous Nervous System Team, Physiology Laboratory, Faculty of Medicine and Pharmacy, Mohammed V University, Rabat, MAR; 3 Acute Medical Unit, Ibn Sina University Hospital, Rabat, MAR; 4 Biostatistics, Clinical, and Epidemiological Research Laboratory, Faculty of Medicine and Pharmacy, Mohammed V University, Rabat, MAR; 5 Medicine, International University of Rabat, Rabat, MAR

**Keywords:** rheumatologists, mri, morocco, non-radiographic axial spondyloarthritis, classification criteria

## Abstract

Introduction

Non-radiographic axial spondyloarthritis (nr-axSpA) is within the spectrum of axial spondyloarthritis (axSpA). The emergence of the nr-axSpA concept, defined by the absence of significant erosive damage to the sacroiliac joints, has prompted numerous initiatives aimed at enhancing the early detection and management of this condition. The aim of the study was to assess the knowledge, attitudes, and practices related to the diagnosis and management of nr-axSpA by rheumatologists in Morocco.

Methods

We conducted a cross-sectional online survey among the rheumatologist community in Morocco. Rheumatologists received via e-mail a structured Google Forms (Google Inc., Mountainview, CA) questionnaire divided into four sections: sociodemographic data of rheumatologists, knowledge, attitudes, and practices related to the diagnosis and treatment management of nr-axSpA.

Results

A total of 110 rheumatologists (mean age of 44±13 years, 77.3% females, median professional experience of 12 years (4, 75; 26.25 years)) participated in the survey (response rate of 25%). Most responders reported a diagnosis delay issue in spondyloarthritis (SpA) (93.6%); 70.9% of rheumatologists incorrectly regarded the 2009 Assessment of Spondyloarthritis International Society (ASAS) classification criteria for axSpA as diagnostic criteria. Rheumatologists' awareness of recommended magnetic resonance imaging (MRI) sequences for detecting sacroiliac joint inflammation and structural changes in SpA varied significantly, from 69.1% to 14.5%. Their knowledge of additional subchondral edema cases in these joints, beyond SpA, ranged from 48.2% to 87.3%. Almost all rheumatologists believed that the use of sacroiliac MRI would contribute to the early diagnosis of axSpA (97.3%) but could also lead to false positive diagnoses, according to 47.3% of rheumatologists; 73.6% believed that incorrectly using the 2009 ASAS classification criteria as diagnostic criteria in nr-axSpA could also result in false-positive diagnoses. In their practice, 2009 ASAS classification criteria were used as diagnostic criteria in axSpA by 39.1% of rheumatologists. Of the total participants, 91.8% indicated that they approach nr-axSpA similarly to radiographic axial spondyloarthritis, with disparities in recommendations of biological therapies.

Conclusion

Our survey provides insight into the current status of nr-axSpA management among Moroccan rheumatologists. It also addresses concerns regarding the risk of false positive diagnoses when using the 2009 ASAS classification criteria for axSpA as diagnostic criteria by rheumatologists and the potential risk of misdiagnosis associated with excessive reliance on MRI, despite its utility for early diagnosis.

## Introduction

Spondyloarthritis (SpA) encompasses a group of inflammatory rheumatic diseases primarily affecting the axial skeleton, sacroiliac joints, and peripheral joints, along with entheses and extra-articular structures. Early recognition and management of SpA are paramount due to its potential for significant disability and impaired quality of life if left untreated. Within the spectrum of SpA, non-radiographic axial spondyloarthritis (nr-axSpA) emerges as a distinct entity, indicating inflammatory sacroiliitis and/or spinal involvement without radiographic evidence of structural damage, akin to ankylosing spondylitis (AS), the prototypical radiographic form [1].

In North Africa, SpA has received less attention compared to rheumatoid arthritis, partly due to the perception that it is rare and relatively benign in certain populations. Moreover, diagnosing SpA is often delayed [2]. The 2009 Assessment of Spondyloarthritis International Society (ASAS) classification criteria for axial spondyloarthritis (axSpA) as diagnostic criteria were devised to aid in identifying individuals likely to have axial SpA for research and clinical trial enrollment. It's noteworthy that these criteria serve as classification tools rather than diagnostic tools [3]. Additionally, a crucial aspect of understanding SpA lies in appreciating its continuum, whereby nr-axSpA may gradually evolve into radiographic axial spondyloarthritis (r-axSpA) over time, underscoring the importance of early intervention to potentially modify disease progression and forestall irreversible damage.

Given the complexity and evolving understanding of SpA, it becomes imperative to assess rheumatologists' comprehension and clinical approach towards nr-axSpA. This investigation aims to improve clinical care and outcomes for individuals with SpA, ultimately enhancing rheumatological practice in managing this multifaceted disease spectrum.

The objective of this study, conducted among Moroccan rheumatologists, is to evaluate their knowledge, attitudes, and practices concerning the diagnostic and therapeutic approaches to nr-axSpA. Additionally, the study sought to investigate how rheumatologists incorporate the 2009 ASAS classification criteria for nr-axSpA and MRI findings into their clinical decision-making processes.

## Materials and methods

Type and population of the study

We conducted a national cross-sectional survey among all Moroccan rheumatologists in the public and private sectors between January and February 2024.

Study eligibility criteria

The inclusion criteria were as follows: all rheumatologists practicing in Morocco, whether in the public, private, or university sectors; and there were no exclusion criteria.

Sampling

This is an exploratory, exhaustive study that targeted all Moroccan rheumatologists in the public and private sectors.

Data collection

We collected the data using the online survey tool Google Forms (Google Inc., Mountainview, CA). Rheumatologists received a clickable link to the questionnaire via e-mail. The e-mail list was provided by the Moroccan Society of Rheumatology (SMR).

The questionnaire was developed based on the knowledge, attitudes, and practices of rheumatologists regarding nr-axSpA, following an extensive literature review. After pilot testing with 10 rheumatologists, the questionnaire was revised and structured into 21 multiple-choice questions.

The questionnaire comprised four comprehensive sections: demographic data, knowledge, attitudes, and practices of rheumatologists.

The demographic data section encompassed participants' sex, age, workplace, and experience, providing essential context for understanding the characteristics of the surveyed rheumatologists.

The knowledge section assessed rheumatologists’ understanding of various aspects related to nr-axSpA diagnosis. This included evaluations of delays in diagnosis, familiarity with terminology, comprehension of the continuum between nr-axSpA and AS, knowledge regarding radiographic diagnosis, familiarity with the 2009 ASAS classification criteria, recommended magnetic resonance imaging (MRI) sequences for reliably detecting inflammation and structural changes in sacroiliac joints in SpA, and regarding additional cases of subchondral edema in the sacroiliac joints beyond SpA.

The attitudes section delved into rheumatologists' perspectives on the nr-axSpA concept, the utilization of MRI in diagnosis, perceptions of the risks associated with misdiagnosis, exploration of factors contributing to diagnostic delays, and solutions for addressing delayed diagnosis in nr-axSpA.

The practice section focused on the modalities of utilizing the 2009 ASAS classification criteria that are used to identify patients with either radiographic or non-radiographic axial spondyloarthritis in clinical practice, the interpretation of sacroiliac MRI findings, and therapeutic approaches for nr-axSpA. This section aimed to elucidate the real-world application of guidelines and recommendations in the management of nr-axSpA by rheumatologists in Morocco.

Statistical analysis

A descriptive analysis of the validated data was performed, with qualitative variables presented as numbers and percentages and quantitative variables expressed as means ± standard deviation (SD) or medians with the interquartile range. Analysis was conducted using IBM SPSS Statistics software for Windows, version 20.0 (IBM Corp., Armonk, NY).

Ethical consideration

The survey received approval from the ethics committee of the University Mohammed V, Rabat, Morocco (Faculty of Medicine and Pharmacy, ethical approval no.: 54/24) and was conducted in accordance with the ethical standards of the 1964 Declaration of Helsinki and its later amendments or comparable standards. Each rheumatologist received an information letter and consent form detailing the purpose and process of the study, along with a clickable link to the survey. Completing the self-administered questionnaire implied consent to use the responses, and all data were analyzed anonymously.

## Results

Out of 440 questionnaires sent, 110 rheumatologists completed and returned the questionnaire, yielding a response rate of 25%. The mean age of the participants was 44 ± 13 years, with 77.3% females. The median professional experience was 12 (4.75; 26.25) years, with 30.9% of participants having practiced for more than 20 years. Among the participants, 42.7% were in the private sector, while 12.7%, 20.9%, and 23.6% were rheumatologists in the hospital-university sector, the public sector, and residents of rheumatology in the university hospital sector, respectively. The demographics of the rheumatologists are presented in Table 1.

**Table 1:** Demographic and clinical data of the participants * Expressed as mean and standard deviation; ** Expressed as n(%)

Characteristics	N = 110
Average age (years) *	44.1 ± 13
Gender**	
Female	85(77.3)
Male	25(22.7)
Place of practice**	
Rheumatologist in the public sector	23(20.9)
Rheumatologist in the university hospital sector	14(12.7)
Rheumatologist in the private sector	47(42.7)
Resident in rheumatology in the university hospital sector	26(23.6)
Experience**	
≤5 years	30(27.27)
>5 years, ≤10 years	22(20)
>10 years, ≤20 years	24(21.81)
>20 years	34(30.9)

Knowledge section results

Almost all rheumatologists confirmed that there is often a delay in the diagnosis of SpA (93.6%) and were aware of the new terminology from the 2009 ASAS classification criteria allowing the distinction between nr-axSpA and r-axSpA. Among the participants, 94.5% considered nr-axSpA as an early stage of axSpA.

Standard X-rays have limitations in sensitivity and also in specificity for axSpA diagnosis, according to 55.5% and 45.5% of responders, respectively. Additionally, 80% considered that detecting structural damage in the sacroiliac joints through standard X-rays is not essential for diagnosis.

The 2009 ASAS classification criteria were considered incorrect as diagnosis criteria in axSpA by approximately two-thirds of rheumatologists (70.9%). In cases of positivity of human leukocyte antigen (HLA) B27 and/or the presence of sacroiliitis on MRI, our responders agreed that the rheumatologist's conviction of the diagnosis was mandatory (88.2%).

**Figure 1:**
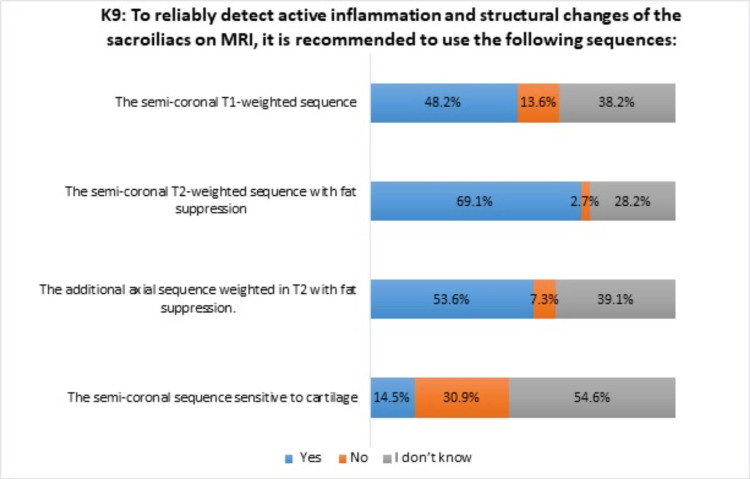
Distribution of responses of rheumatologists to the ninth statement (K9) in the knowledge section

Accurate knowledge regarding the four recommended MRI sequences for reliably detecting inflammation and structural changes in sacroiliac joints in spondyloarthritis varied significantly, ranging from 69.1% to 14.5%. Notably, awareness was particularly deficient regarding the semi-coronal sequence sensitive to cartilage (Figure 1).

**Figure 2:**
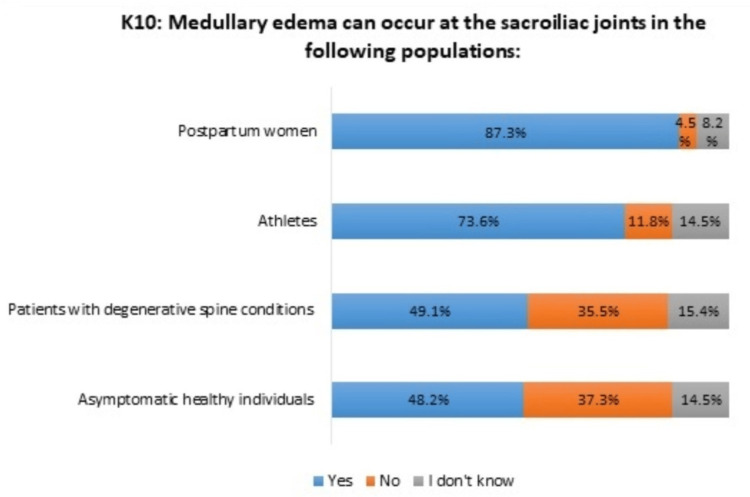
Distribution of responses of rheumatologists to the tenth statement (K10) in the knowledge section

Insufficient knowledge was noticed regarding additional cases of subchondral edema in the sacroiliac joints beyond SpA, particularly its potential presence in asymptomatic healthy individuals and patients with degenerative spine conditions (Figure 2).

Attitudes section results

Nearly all rheumatologists concurred that the emergence of the nr-axSpA concept and the utilization of sacroiliac MRI would enhance early diagnosis, as reported by 96.4% and 97.3% of respondents, respectively. However, the implementation of sacroiliac MRI could potentially result in false-positive diagnoses, according to 47.3% of respondents.

Two-thirds of rheumatologists (73.6%) believed that incorrectly using the 2009 ASAS classification criteria as diagnostic criteria in nr-axSpA could also result in false-positive diagnoses. Table 2 presents rheumatologists' perceptions regarding factors contributing to the delayed diagnosis of axSpA.

**Table 2:** Distribution of rheumatologists' responses to the fifth Statement (A5) in the attitudes section regarding contributing factors to the delayed diagnosis of axial spondyloarthritis MRI: magnetic resonance imaging; NSAIDs: non-steroidal anti-inflammatory drugs

A5 : I believe that the delayed diagnosis of axial spondyloarthritis would be linked to the following factors:	Effective (n)	Percentage (%)
Diagnostic errors in axial spondyloarthritis	103	93.6
Challenges in accessing specialized care provided by rheumatologists	96	87.2
Lack of diagnostic criteria for non-radiographic axial spondyloarthritis	83	75.4
Delayed onset of radiographic signs	91	82.7
The inconsistent nature of inflammatory syndrome	98	89.1
Insufficient awareness of non-radiographic spondyloarthritis among rheumatologists	81	73.6
Patient difficulties accessing MRI	104	94.5
Issues related to protocols for conducting and interpreting sacroiliac MRI	102	92.7
Self-medication of patients with NSAIDs	104	94.5

According to the rheumatologists, various solutions were proposed to address the issue of delayed diagnosis of nr-axSpA, as presented in Table 3.

**Table 3:** Rheumatologists' responses to the sixth statement in the attitudes section regarding proposed solutions for addressing delayed diagnosis in non-radiographic axial spondyloarthritis HLA: human leukocyte antigen

A6: To address the issues of delayed diagnosis of non-radiographic axial spondyloarthritis, I propose the following solution(s):	Effective (n)	Percentage (%)
Increase public awareness of spondyloarthritis	106	96.3
Conduct ongoing training sessions on spondyloarthropathies for general practitioners	100	90.9
Implement continuous training programs on non-radiographic spondyloarthritis	109	99.1
Standardize the patient pathway in case of suspected non-radiographic spondyloarthritis	109	99.1
Facilitate access for patients with suspected non-radiographic axial spondyloarthritis to HLA-B27 typing	105	95.4
Facilitate access for patients with suspected non-radiographic axial spondyloarthritis to sacroiliac MRI	110	100
Strengthen collaboration between rheumatologists and radiologists	109	99.1

Practices section results

In their practice, 39.1% of rheumatologists used the 2009 ASAS classification criteria as diagnostic criteria. Rheumatologists interpreted subchondral edema in the sacroiliac joints on MRI based on the patient's clinical context (90%), the location of the subchondral edema (84.5%), and the ASAS definition for sacroiliitis on MRI in SpA (88.2%). However, only 10% of rheumatologists considered the presence of subchondral edema in the sacroiliac joints on MRI as pathognomonic of nr-axSpA. The majority of rheumatologists (91.8%) indicated that they manage nr-axSpA in the same way as radiographic axial spondyloarthritis. However, there were varied approaches to recommending biological treatment for patients with nr-axSpA, as presented in Table 4.

**Table 4:** Rheumatologists' responses to the fifth statement (P5) in the practice section BASDAI: Bath Ankylosing Spondylitis Disease Activity Index; ASDAS: Ankylosing Spondylitis Disease Activity Score; CRP: C-reactive protein; MRI: magnetic resonance imaging

P5: In cases of non-radiographic axial spondyloarthritis and in the event of an inadequate response to NSAIDs, biologic therapy is recommended:	Effective (n)	Percentage (%)
In the presence of clinical activity (BASDAI ≥4 or ASDAS ≥2) and objective signs of inflammation: elevated biological markers (CRP) and/or on MRI (sacroiliitis)	110	100
In the presence of clinical activity (BASDAI ≥4 or ASDAS ≥2), even in the absence of objective signs of inflammation: normal biological markers (CRP), and/or no sacroiliitis on MRI	43	39.1
In the presence of objective signs of inflammation: elevated biological markers (CRP) and/or on MRI (sacroiliitis), even in the absence of signs of clinical activity (BASDAI <4 or ASDAS <2)	40	36.3
Even in the absence of signs of clinical activity (BASDAI <4 or ASDAS <2) and objective signs of inflammation: elevated biological markers (CRP) and/or on MRI (sacroiliitis)	11	10

## Discussion

Our study provides a comprehensive overview of the knowledge, attitudes, and practices of rheumatologists in Morocco regarding nr-axSpA. Additionally, we investigated the obstacles and deficiencies encountered by rheumatologists in integrating the 2009 ASAS classification criteria for nr-axSpA and MRI into their clinical practice.

Patients with axSpA are categorized into two types based on the presence of definite structural changes in the sacroiliac joints on conventional radiographs. Those fulfilling the modified New York (mNY) criteria are diagnosed with r-axSpA or AS, while those not meeting the criteria but showing sacroiliitis on MRI are diagnosed with nr-axSpA [4].

The 2009 axSpA classification criteria by ASAS have been crucial in understanding nr-axSpA. These criteria, emphasizing clinical or imaging parameters such as HLA-B27 status and symptoms like inflammatory back pain, allow for earlier identification and intervention in patients with suggestive symptoms but without definitive radiographic changes [4]. However, there is ongoing debate regarding the inclusion of patients based solely on clinical parameters, such as a positive HLA-B27 test, without objective evidence of axial inflammatory disease.

The ASAS criteria are designed to facilitate early management. Awareness regarding the delayed diagnosis of SpA is evident among the majority of responding rheumatologists. Previous studies have highlighted a significant delay in diagnosis in Morocco, with substantial findings revealing a burden for both patients and society [5-7]. According to Rkain et al., this delay leads to considerable direct and indirect costs and noticeably affects the quality of life of patients [8].

The delayed diagnosis of AS is a common issue that can have significant consequences for a patient's health. Studies have shown that this delay can lead to functional deterioration, reduced quality of life, and increased long-term healthcare costs. Moreover, delayed diagnosis may compromise treatment effectiveness and worsen structural damage, highlighting the importance of early disease identification to optimize clinical outcomes [6].

Protopopov et al. reported progression from nr-axSpA to r-axSpA over a two- to 10-year period, with rates ranging from 10% to 40%. This underscores the dynamic nature of the disease. Similar findings have been reported by other authors [9].

Hammoudeh et al. and Slimani et al. have also shed light on the challenges surrounding the diagnosis and management of axSpA in North Africa and the Middle East. Both papers highlight the barriers faced by healthcare professionals in these regions, such as limited access to diagnostic tools, variations in disease presentation, and cultural influences on patient care. They stress the importance of early diagnosis and tailored management strategies to improve outcomes for patients with axSpA [2, 7].

In our study population, diagnostic delays were primarily attributed to difficulties in accessing specialized care from rheumatologists, a sentiment echoed by our respondents. Challenges in obtaining timely appointments and long waiting times were commonly reported, highlighting the limited availability of rheumatology services in certain regions. Furthermore, patients faced significant hurdles in accessing MRI scans, which are essential for accurate diagnosis and disease monitoring in spondyloarthritis.

The integration of MRI into rheumatological practice has revolutionized the early detection of inflammation in axSpA, allowing for timely intervention before significant structural damage occurs [10,11]. However, the recognition of sacroiliitis on MRI poses challenges. According to a German study involving rheumatologists, a significant proportion of radiologists do not assess sacroiliac joint MRI images according to established criteria, underscoring the need for standardized evaluation methods [12].

To reliably detect active inflammation and structural changes in the sacroiliac joints, the radiologists of the European Society of Skeletal Radiology (ESSR) Arthritis Subcommittee [13] and the ASAS and the Spondyloarthritis Research and Therapy Network (SPARTAN) recommend four specific sequences to reliably detect inflammation and structural changes in the sacroiliac joints: axial T1-weighted and short TI inversion recovery (STIR) sequences, coronal STIR sequences, and axial T1-weighted sequences with fat suppression. Our study revealed a lack of familiarity among rheumatologists with certain recommended sequences, particularly the semi-coronal sequence sensitive to cartilage.

Furthermore, nearly half of the rheumatologists surveyed expressed concerns that sacroiliac MRI could lead to a misdiagnosis of nr-axSpA. Subchondral bone edema is considered a pathognomonic sign of specific sacroiliitis in SpA according to the ASAS definition for active sacroiliitis, developed in 2009 [14] and continually refined [15]. However, this edema can also be observed in various other conditions, including degenerative diseases, and in healthy individuals, particularly postpartum women. [16,17].

The lack of awareness among surveyed rheumatologists regarding the potential for false positives in MRI interpretation underscores the importance of ongoing education and training in medical imaging, in collaboration with radiologists. This would ensure consistent interpretation of results and better recognition of situations that may present false appearances of sacroiliitis.

Our study underscores the importance of ongoing education and interdisciplinary collaboration between rheumatologists and radiologists to ensure accurate interpretation of sacroiliac MRI and optimal management of patients with inflammatory axial pathologies. These efforts are critical for improving early diagnosis and clinical outcomes in this field.

Our findings reveal a notable divergence in the utilization of the 2009 ASAS classification criteria for SpA among rheumatologists. While a significant proportion directly apply these criteria as diagnostic tools, a larger majority utilize them as guiding principles without relying on them as strict diagnostic criteria. This disparity underscores the varied interpretations and applications of the ASAS criteria within clinical practice.

It’s crucial to emphasize that the ASAS criteria are primarily intended for classification rather than diagnostic purposes [3,18]. As such, their application as diagnostic criteria may lead to misinterpretations and potential misdiagnoses [19]. While the ASAS criteria provide valuable frameworks for identifying patients who may belong to the SpA spectrum, they do not replace the need for comprehensive clinical assessment and expertise in rheumatological diagnosis.

Therefore, our findings underscore the importance of adhering to the intended purpose of the ASAS criteria as classification tools within clinical practice. Rheumatologists should utilize these criteria judiciously, supplementing them with thorough clinical evaluation, including patient history, physical examination, and additional diagnostic tests, to ensure accurate and timely diagnosis of SpA and its subtypes. This approach highlights the need for ongoing education and training to enhance clinicians’ understanding and application of classification criteria in rheumatological practice.

The key goals of the treatment of axSpA include improving symptoms, decreasing inflammation, improving function and quality of life, and preventing irreversible skeletal damage and new bone formation in the spine.

It is crucial to consider objective indicators of inflammatory activity, such as MRI findings or elevated C-reactive protein (CRP) levels, when initiating biological treatments for patients with axSpA lacking radiographic sacroiliitis. Several studies have highlighted the importance of discerning between inflammatory and non-inflammatory sources of symptoms in nr-axSpA. Without active signs of inflammation, patient symptoms, particularly back pain, may not be attributable to inflammation, even in cases where nr-axSpA is diagnosed [20].

The management approach for nr-axSpA largely aligns with that of radiographic forms, albeit with complexities in defining disease activity. However, research suggests that non-radiographic forms lacking objective signs of inflammation, as detected by MRI or CRP, may not demonstrate a significant therapeutic response to tumor necrosis factor inhibitors compared to placebo [21-23]. This lack of response may partly be due to misinterpretations and misdiagnoses, where patients without true inflammatory axSpA might be incorrectly classified under nr-axSpA. In the absence of signs of inflammatory activity, it is highly probable that the patient's symptoms (primarily back pain) are not related to inflammation, even if the diagnosis of nr-axSpA is correct. Therefore, the correct diagnosis and objective evidence of inflammatory activity (active inflammation in the sacroiliac joints or spine observed on MRI, elevated CRP) are the main factors that define a favorable treatment response in axSpA, including the non-radiographic stage.

Furthermore, considering the concept of 'difficult-to-treat' SpA sheds light on patients who do not respond adequately to standard treatments. These patients may present with a spectrum of challenges, including misdiagnosis, intrinsic resistance to therapy, or comorbidities such as fibromyalgia, which can explain, in some cases, the remaining pain and treatment failures. 

In accordance with the guidelines from the Moroccan SMR for the therapeutic management of SpA [24], biological treatments are recommended for nr-axSpA patients with an inadequate response to non-steroidal anti-inflammatory drugs, along with specific criteria including Bath Ankylosing Spondylitis Disease Activity Index (BASDAI) ≥4 or Ankylosing Spondylitis Disease Activity Score (ASDAS) ≥2.1 and positive CRP and/or inflammatory signs on MRI.

Our study findings indicate that a vast majority of rheumatologists adhere to these recommendations, with only a small percentage suggesting biologic therapy for nr-axSpA patients in the absence of clinical or objective signs of inflammation, such as elevated CRP or sacroiliitis on MRI.

The broader healthcare landscape in North Africa presents challenges in accessing specialized care and resources for early diagnosis. A survey highlighting these challenges [7] reflects systemic issues within the healthcare system, including disparities in healthcare infrastructure, shortages of healthcare professionals, and limited funding for rheumatology services. These systemic barriers further contribute to delays in diagnosis and treatment initiation for patients with SpA.

Improving the management of nr-axSpA necessitates ongoing efforts in education and training to enhance understanding and proficiency in diagnosing and treating this condition. There is a crucial need for continued education among rheumatologists and radiologists to standardize MRI protocols for inflammatory back pain. Additionally, fostering greater collaboration between these specialists is imperative to ensure consistent and effective interpretation of imaging findings. Furthermore, efforts should be directed towards educating general practitioners to facilitate early referral of suspected cases of SpA to rheumatologists, as well as streamlining patient pathways to enhance accessibility to HLA-B27 testing and MRI imaging. These endeavors are pivotal in improving patient outcomes and reducing the burden of nr-axSpA on individuals and healthcare systems.

Our study possesses several notable strengths. It marks the first comprehensive national survey conducted among Moroccan rheumatologists, offering profound insights into the management of nr-axSpA throughout the country. Through the meticulous assessment of knowledge, attitudes, and practices, our study provides a holistic understanding of the prevailing landscape surrounding nr-axSpA among rheumatologists.

Furthermore, our findings address pivotal issues such as diagnostic delays, the efficacy of MRI in early detection, and the challenges associated with applying classification criteria. These insights offer invaluable guidance for clinical practice, potentially enhancing the quality of care provided to patients with nr-axSpA.

We believe that our study significantly contributes to the existing body of literature on nr-axSpA, particularly in regions like Morocco that have been underrepresented in research, by filling gaps in knowledge and raising awareness. Our study serves as a vital step towards improving the understanding and management of nr-axSpA, laying the groundwork for future research and interventions aimed at improving patient care and outcomes.

However, our study also has its limitations. Despite our efforts to obtain a representative sample, the size of 110 rheumatologists may not fully capture the diversity of practices and perspectives within the country. Moreover, reliance on self-reported data introduces the potential for bias or inaccuracies, especially concerning practices and attitudes. Lastly, the cross-sectional design of our study restricts our ability to establish causality or monitor changes in knowledge, attitudes, and practices over time.

## Conclusions

This knowledge, attitudes, and practices survey was conducted among Moroccan rheumatologists on nr-axSpA and holds paramount significance in understanding and enhancing the management of this condition. Our study findings underscore the crucial significance of addressing the prevailing diagnostic challenges encountered by patients with axSpA. It is imperative to recognize and rectify the knowledge gaps among rheumatologists, particularly concerning recommended MRI sequences and the proper application of classification criteria for nr-axSpA. Establishing professional networks dedicated to the management of nr-axSpA could significantly enhance diagnostic and therapeutic approaches. Moreover, advocating for improved accessibility to early diagnostic tools, such as MRI, is paramount to ensuring timely and accurate diagnosis, ultimately leading to better outcomes for patients with this condition.
